# Upregulation of heme oxygenase-1 expression by curcumin conferring protection from hydrogen peroxide-induced apoptosis in H9c2 cardiomyoblasts

**DOI:** 10.1186/s13578-017-0146-6

**Published:** 2017-04-21

**Authors:** Xiaobo Yang, Hong Jiang, Yao Shi

**Affiliations:** 1 Department of Ophthalmology, Union Hospital, Tongji Medical College, Huazhong University of Science and Technology, Wuhan, 430022 China; 2 Department of Neonatology, The Central Hospital of Wuhan, Tongji Medical College, Huazhong University of Science and Technology, Wuhan, 430014, China

**Keywords:** Curcumin, Cardiomyocyte apoptosis, Oxidative stress, Heme oxygenase-1, PI3K/Akt

## Abstract

**Background:**

Curcumin is a major constituent of rhizomes of Curcuma longa that elicits beneficial effects for oxidative damage. The aim of this study was to investigate whether curcumin could attenuate hydrogen peroxide (H_2_O_2_)-induced apoptosis in H9c2 cardiomyoblasts and the underlying mechanisms.

**Results:**

The present study showed that exposure of H9c2 cells to H_2_O_2_ caused a significant increase in apoptosis as evaluated by flow cytometry analysis and the pretreatment of curcumin protected against H_2_O_2_-induced apoptosis. Exposure of cells with curcumin caused a dose-dependent induction of heme oxygenase-1 (HO-1) protein expression. Curcumin also decreased the cleaved caspase-3 (CC3) protein expression level and increased the Bcl-2/Bax ratio in H_2_O_2_-stimulated H9c2 cells. ZnPP-IX, a HO-1 inhibitor, partly reversed the anti-apoptotic effect of curcumin. Further, LY294002, an inhibitor of PI3K, partially reversed the effect of curcumin on HO-1 protein induction, leading to the attenuation of curcumin-mediated apoptosis resistance.

**Conclusion:**

These results demonstrated that the anti-apoptotic function of curcumin required the upregulation of HO-1 protein through the PI3K/Akt signaling pathway. Curcumin might be used as a preventive and therapeutic agent for treatment of cardiovascular diseases associated with oxidative stress.

## Background

Oxidative stress-induced apoptosis has long been implicated in the pathogenesis of cardiovascular diseases such as myocardial ischemic injury and infarction [1, 2]. Oxidative damage, mediated by reactive oxygen species (ROS) which can be generated following cell lysis, oxidative burst, or the presence of an excess of free transition metals, can attack proteins, DNA, and membrane lipids, thereby leading to the loss of cell integrity, enzyme function, and genomic stability [3, 4]. Therefore, therapeutic intervention targeting the apoptosis is a reasonable strategy for the treatment of cardiovascular diseases.

Curcumin, a major component of turmeric powder extracted from the rhizomes of the plant Curcuma longa, has been applied for centuries in indigenous medicine to treat various diseases [5]. This bioactive phytochemical is a potent inhibitor of tumor promotion and possesses anti-inflammatory and anti-oxidative activities [6]. In addition, curcumin seems to be, even at relatively low concentrations, an effective anti-apoptotic agent [7]. One study reported that curcumin attenuated peroxynitrite-induced apoptosis in primary cultured rat spiral ganglion neurons [8], and another study demonstrated that curcumin had the potential to protect experimental autoimmune myocarditis [9]. Nevertheless, the possible protective effect of curcumin on the toxicity in cardiomyoblasts has not been tested in vitro. Furthermore, the precise mechanism underlying this response is still unclear.

Heme oxygenase-1 (HO-1), which is the rate-limiting enzyme responsible for the degradation of heme into free ferrous iron, carbon monoxide (CO) and bilirubin, exerts cytoprotective effects in various diseases [10, 11]. Recent experimental evidence indicated that increased HO-1 production provided cellular protection against oxidative injury induced by ischemia/reperfusion [12] or the use of hydrogen peroxide (H_2_O_2_) [13, 14]. Protein kinase B (PKB, Akt), one of the most important downstream target kinases of phosphoinositide 3-kinase (PI3K), is an important signaling molecule activated by anti-apoptotic agents [15], while extracellular signal-regulated kinases (ERKs) mediate another important signaling pathway involved in anti-apoptotic effects [16]. Several studies reported that HO-1 provided protection against various forms of stress through the activation of the PI3K/Akt or ERK1/2 signaling pathways [17, 18].

The H9c2 cell line, derived from the embryonic BDIX rat heart ventricle, is considered a close surrogate for cardiomyocytes and has been proven to be ideal for signal transduction studies [19]. H_2_O_2_, as one of the main ROS, could cause DNA damage and lipid peroxidation and has been widely used to induce apoptosis in various cell types [20]. In this study, H_2_O_2_ was used to induce apoptosis in H9c2 cells, as it is a well-established model to study oxidative stress-induced cardiomyocyte apoptosis [21, 22]. Here we aimed to investigate the anti-apoptotic effect of curcumin in H_2_O_2_-stimulated H9c2 cells and to explore the role of HO-1 and its associated signaling pathways.

## Methods

### Chemicals and reagents

Curcumin, Zine protoporphyrin-IX (ZnPP-IX, a HO-1 inhibitor), dimethyl sulfoxide (DMSO), H_2_O_2_ and methyl thiazolyl tetrazolium (MTT) were from Sigma Chemical. LY294002 (a PI3K inhibitor) and rabbit polyclonal antibodies specific for total ERK1/2 (t-ERK1/2), phospho-ERK1/2 (p-ERK1/2), total Akt (t-Akt), phospho-Akt (p-Akt, serine 473), Bcl-2, Bax, cleaved caspase-3 (CC3) and GAPDH were from cell signaling. Rabbit polyclonal antibodies specific for HO-1 were obtained from Stressgen Bioreagents.

### Cell culture

H9c2 cardiomyoblasts from the American Type Culture Collection (ATCC, CRL-1446) were maintained in DMEM supplemented with 10% heat-inactivated fetal bovine serum, 100 U/mL penicillin and 100 μg/mL streptomycin in a humid atmosphere of 5% CO_2_ and 95% air at 37 °C. Cells were regularly passaged and subcultured to 90% confluence before experimental procedures. Curcumin dissolved in DMSO was diluted with low-serum medium (1% FBS/DMEM) to the final concentrations before use. The final concentration of DMSO in the incubation mixture was not more than 0.1% (v/v).

### Cell viability assay

Cell viability was assessed by MTT assay. Briefly, the H9c2 cells subcultured in 96-well plates at 1 × 10^4^ cells/well were incubated with the test chemicals for indicated time period. Then 5 mg/mL MTT was added to the culture media and cells were incubated further for an additional 4 h. After this incubation, the formed formazan was solubilized by adding DMSO, and optical density of the solubilized cell extract was measured at 490 nm using a microplate reader. The reduction in optical density was considered being the decrease in cell viability.

### Annexin-V FITC/PI assay

Apoptosis was detected using an Annexin-V FITC/PI detection kit according to the manufacturer’s directions (KeyGEN, Nanjing, China). The cells were digested with 0.25% trypsin, washed with ice-cold PBS and resuspended in binding buffer (5 × 10^5^  cells/mL). Then, the cells were centrifuged at 1000*g* for 5 min at 4 °C. After the supernatant had been discarded, 500 μL of binding buffer, 5 μL of annexin-V-FITC and 5 μL of propidium iodide were added to the cell suspension. After mixing gently, the suspensions were incubated for 15 min at room temperature without light. Finally, the cells were analyzed by flow cytometry (BD LSRII; BD Biosciences).

### Western blot analysis

Cells were lysed in ice-cold cell lysis buffer. The protein concentration was determined using BCA method. Protein was separated by SDS-PAGE, and then transferred onto polyvinylidene difluoride membrane. The membranes were blocked in TBS-T with 5% (w/v) skim milk at room temperature for 2 h, followed by overnight incubation at 4 °C with primary antibodies diluted in TBS-T. After washing in TBS-T, the membranes were incubated for 1 h with a horseradish peroxidase-conjugated secondary antibody diluted in TBS-T. After washing once more in TBS-T, the labeled protein was detected using enhanced chemiluminescence reagents and exposed to film. The intensity of the bands was analyzed with Alpha Ease FC image software.

### Statistical analysis

All data represented the mean of samples from three independent experiments. Results were presented as mean and standard deviation (mean ± SD). Statistical significance was determined by one-way ANOVA followed by Student–Newman–Keuls test for comparison of several groups. A *p* value less than 0.05 was considered being statistically significant.

## Results

### Curcumin reduced H_2_O_2_-induced cell toxicity

**Fig. 1:**
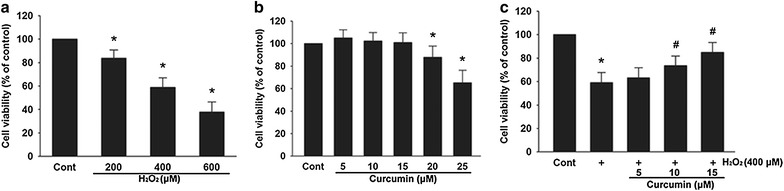
Curcumin reduced H_2_O_2_-induced cell toxicity. Cell viability was examined using the MTT assay. **a** Effect of H_2_O_2_ on cell viability. Cells were treated with 200–600 μM of H_2_O_2_ for 3 h. **b** Effect of curcumin on cell viability. Cells were treated with 5–25 μM of curcumin for 24 h. **c** Curcumin protected the cells from H_2_O_2_-induced cytotoxicity in a dose-dependent manner. After pretreated with 5–15 μM of curcumin for 12 h, the cells were washed and incubated with 400 μM H_2_O_2_ for 3 h. Data were presented as mean ± SD (n = 3). *p < 0.05 vs. Cont (control), ^#^ *p* < 0.05 vs. H_2_O_2_

As shown in Fig. 1a, 200–600 μM H_2_O_2_ reduced the cell viability in a dose-dependent manner. In the presence of 400 and 600 μM of H_2_O_2_, the percentage of viable cells was reduced to 58.92 ± 8.02 and 37.76 ± 8.54% of the control, respectively (*p* < 0.05). Then we evaluated whether curcumin was cytotoxic to H9c2 cells. As shown in Fig. 1b, cell viability was not significantly affected by treatment with increasing doses of curcumin up to 15 μM compared to that of the control group. However, a significant decrease in cell viability was observed in cells treated with 20 and 25 μM curcumin (87.88 ± 9.85 and 65.3 ± 10.94% of the control, *p* < 0.05). Next, we tested whether the pretreatment with curcumin was able to protect against H_2_O_2_-induced cytotoxicity. As shown in Fig. 1c, pretreatment with 10 and 15 μM of curcumin significantly increased the cell viability to 73.61 ± 8.14 and 84.93 ± 8.41% of the control, respectively. Our results indicate that curcumin may have protective role against H_2_O_2_-induced cell death.

### Curcumin increased HO-1 protein expression

**Fig. 2:**
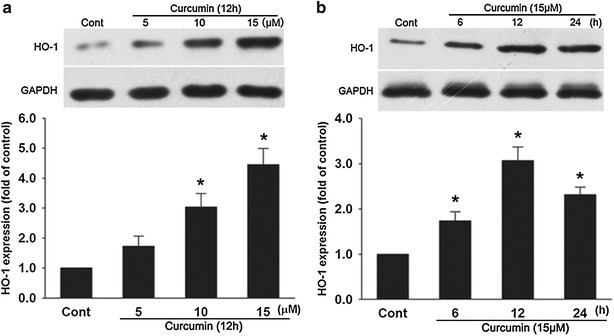
Curcumin increased HO-1 protein expression. **a** Cells were incubated with 5–15 μM of curcumin for 12 h as indicated. **b** Cells were incubated with 15 μM of curcumin for the indicated amounts of time. HO-1 protein expression was determined by western blot analysis. Data were presented as mean ± SD (n = 3). *p < 0.05 vs. Cont

Curcumin treatment for 12 h increased HO-1 protein expression in a dose-dependent manner (Fig. 2a). Curcumin (15 μM) induced a significant increase of HO-1 protein expression for the 3-time points tested, with a maximum of 3.06 ± 0.31-fold increase after the 12 h treatment (Fig. 2b).

### The anti-apoptotic effect of curcumin was reversed by ZnPP-IX

**Fig. 3:**
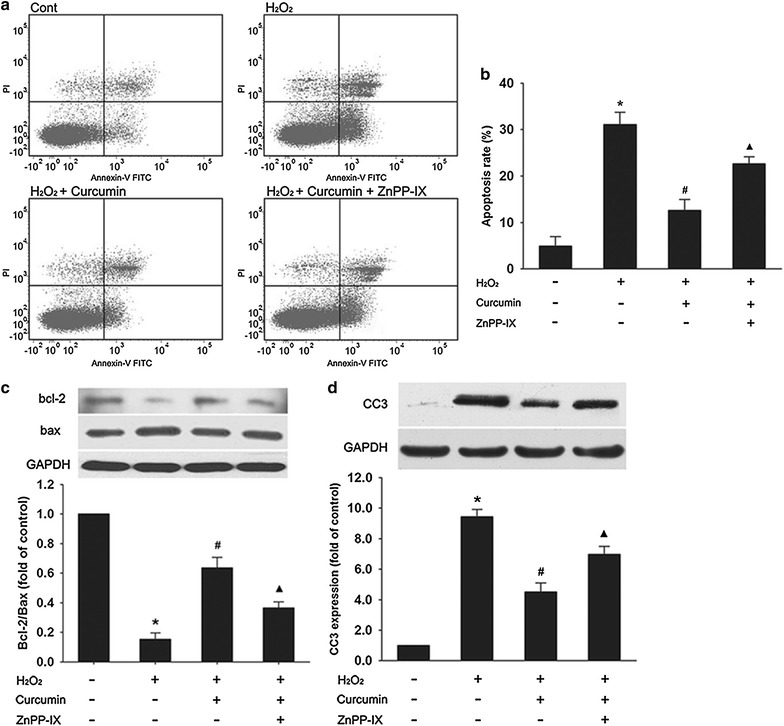
The anti-apoptotic effect of curcumin was partly reversed by ZnPP-IX. After pretreated with 15 μM of curcumin for 12 h in the absence or presence of 10 μM ZnPP-IX, the cells were washed and incubated with 400 μM H_2_O_2_ for 3 h. **a**, **b** Percentage of apoptotic cells was detected by flow cytometry analysis using Annexin-V FITC/PI staining. Apoptotic cells included Annexin V (+)/PI (−) and Annexin V (+)/PI (+) cells. **c**, **d** The Bcl-2, Bax, and CC3 protein expression were determined by western blot analysis. The Bcl-2/Bax ratio was calculated. Data were presented as mean ± SD (n = 3). *p < 0.05 vs. control, ^#^ *p* < 0.05 vs. H_2_O_2_, ^▲^ *p* < 0.05 vs. H_2_O_2_ + curcumin

As shown in Fig. 3a, b, 400 μM of H_2_O_2_ led to a significant increase in apoptosis in H9c2 cells compared with the control group, and apoptosis was decreased markedly by curcumin. The anti-apoptotic effect of curcumin was notably reversed by ZnPP-IX. We next showed that H9c2 cells subjected to H_2_O_2_ had decreased Bcl-2/Bax ratio compared with the control group, while curcumin pretreatment increased the Bcl-2/Bax ratio compared with the H_2_O_2_ group. Again, this effect of curcumin was partly blocked by ZnPP-IX (Fig. 3c). Furthermore, western blot analysis also showed that H_2_O_2_ caused a significant increase in CC3 levels compared with the control which is reduced by the pretreatment with curcumin. Co-incubation with ZnPP-IX partly negated this effect of curcumin (Fig. 3d).

### Curcumin enhanced phosphorylation of Akt but had no influence on ERK1/2 phosphorylation

**Fig. 4:**
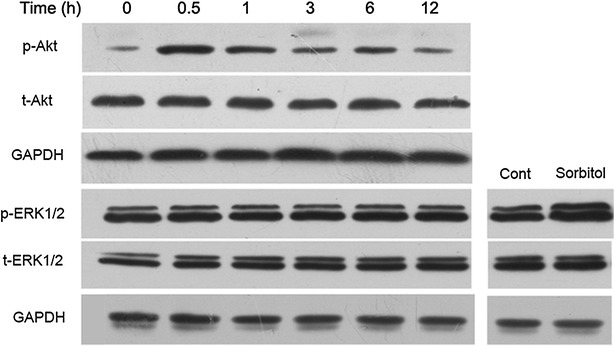
Curcumin enhanced phosphorylation of Akt but had no influence on ERK1/2 phosphorylation. Cells were treated with 15 μM curcumin for the indicated times. The expression levels of p-Akt, t-Akt, p-ERK1/2 and t-ERK1/2 were measured by western blot analysis. Cell extracts from H9c2 cells stimulated by osmotic shock (0.5 M sorbitol, 30 min) served as a positive control. Representative blots of three independent experiments were shown

As shown in Fig. 4, the levels of p-Akt increased remarkably in the first 30 min and then began to decrease continuously in the following hours, while curcumin had no significant influence on p-ERK1/2 at any time point tested. In addition, total levels of Akt and ERK1/2 did not change significantly among these treatments.

### Influence of LY294002 on apoptosis and HO-1 expression

**Fig. 5:**
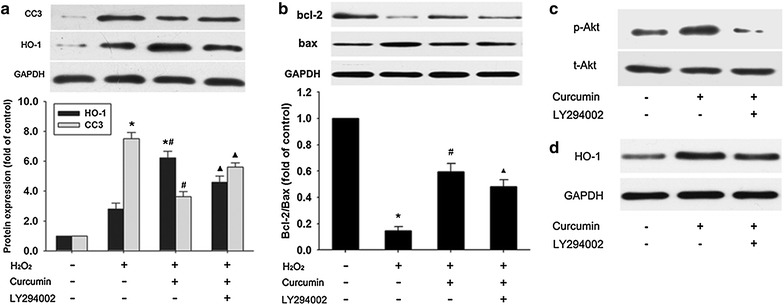
Influence of LY294002 on apoptosis and HO-1 expression. **a**, **b** After pretreated with 15 μM of curcumin for 12 h in the absence or presence of 50 μM of LY294002, the cells were washed and incubated with 400 μM H_2_O_2_ for 3 h. **c** Cells were treated with 15 μM curcumin for 30 min in the absence or presence of 50 μM LY294002, which was added 1 h before curcumin. **d** Cells were incubated with 15 μM of curcumin for 12 h in the absence or presence of 50 μM of LY294002. The CC3, Bcl-2, Bax, HO-1, p-Akt and t-Akt protein expression were determined by western blot analysis. The Bcl-2/Bax ratio was calculated. Data were presented as mean ± SD (n = 3). *p < 0.05 vs. control, ^#^ *p* < 0.05 vs. H_2_O_2_, ^▲^ *p* < 0.05 vs. H_2_O_2_ + curcumin

To determine whether the activation of the PI3K/Akt pathway by curcumin is instrumental to the survival of H9c2 cells by modulating HO-1 expression, we tested the effects of LY294002 (an inhibitor of PI3K) on the protein expression of CC3 and HO-1. As shown in Fig. 5a, curcumin decreased the CC-3 protein expression levels compared with the H_2_O_2_ group, but this effect was largely negated by LY294002. The increase of HO-1 protein expression induced by curcumin was also partly abolished by LY294002. Furthermore, curcumin increased the Bcl-2/Bax ratio compared with the H_2_O_2_ group. And this effect of curcumin was also partially blocked by LY294002 (Fig. 5b). As expected, Akt phosphorylation enhanced by curcumin was completely reduced by LY294002 (Fig. 5c). Co-incubation with LY294002 partly negated the increase of HO-1 induced by curcumin (Fig. 5d).

## Discussion

H_2_O_2_ is a strong oxidant that can cause a marked decrease in cell viability. The present study confirmed that treating H9c2 cells with H_2_O_2_ resulted in a dose-dependent viability loss. Curcumin is a hormetic compound, at higher doses it is cytotoxic, but at lower doses, it is implicated in cellular adaptive stress responses [23]. Our study showed that administration of curcumin at higher doses (20 and 25 μM) for 24 h induced cell death, whereas curcumin lower than 15 μM (including 15 μM) were nontoxic to H9c2 cells. We then investigated the protective effect of curcumin against H_2_O_2_-induced cell toxicity by MTT assay. The results showed that curcumin protected H9c2 cells from H_2_O_2_-induced cytotoxicity in a dose-dependent manner.

We have shown that H9c2 cells incubation with 400 μM of H_2_O_2_ decreased the cell viability about 40% in comparison to the control. Moreover, typical features of apoptosis such as an increase of phosphatidylserines externalization, an elevated CC3 expression [24] and a decreased Bcl-2/Bax ratio [25] indicate that the cell death observed in the cell viability assay is mainly of apoptotic nature. Recently, curcumin was shown to be implicated in the suppression of apoptosis in various cell types such as vascular smooth muscle cells [26] and renal proximal tubular cells [27]. Our study demonstrated for the first time that H_2_O_2_-induced apoptosis of H9c2 cells was significantly inhibited by curcumin pretreatment.

Pharmacological and genetic induction of HO-1 has been shown to exert an anti-apoptotic effect in various cardiovascular diseases [28, 29]. A previous study demonstrated that HO-1 was upregulated in endothelial cells [30] and skin fibroblast cells [31] by curcumin in vitro, and here we showed that curcumin induced HO-1 protein expression in a dose-dependent manner in H9c2 cells. In addition, the anti-apoptotic effect of curcumin was demonstrated to be partly attributed to the induction of HO-1 because the inhibitor of HO-1 (ZnPPIX) markedly reversed the protection of curcumin, as revealed by a decrease of Bcl-2/Bax ratio, and an increase of CC3 protein expression and apoptotic cells. These results suggest that the induction of HO-1 may play a significant role in mediating the anti-apoptotic effect of curcumin in H_2_O_2_-stimulated H9c2 cells.

Since PI3K/Akt and ERK1/2 are the common signaling pathways for the modulation of HO-1 expression [17, 18], the influence of curcumin on the phosphorylation of Akt and ERK1/2 was measured. In our study, curcumin activated the PI3K/Akt pathway, but not the ERK1/2 pathway. These effects of curcumin are consistent with previous evidence using rat aortic vascular smooth muscle cells [26]. This means that it is the phosphorylation of Akt but not ERK1/2 involved in curcumin-mediated protection. Although most studies showed the PI3K/Akt pathway participated in the regulation of HO-1 expression, the role of Akt phosphorylation in HO-1 activation still remained controversial. For instance, in agreement with our data, pharmacological activation of the PI3K/Akt pathway by carnosol (a constituent of the herb of rosemary), which led to the induction of HO-1 protein, efficiently protected rat pheochromocytoma PC12 cells against oxidative stress [32]. However, piceatannol which is an anti-inflammatory and anti-proliferative plant-derived stilbene elevated HO-1 protein levels in bovine aortic endothelial cells via PKC and tyrosine kinase pathways, but not the PI3K/Akt pathway [33]. A possible explanation for these different findings could be that the mechanisms of HO-1 activation induced by various chemicals may differ significantly in different cell types. Our results are consistent with the requirement of Akt phosphorylation for the upregulation of HO-1 by curcumin because upregulation of HO-1 expression induced by curcumin was partly blocked by LY294002. In addition, LY294002 also partially reversed the anti-apoptotic effect of curcumin. These results suggested that the induction of HO-1 through the PI3K/Akt pathway was critically involved in curcumin-mediated apoptosis resistance.

ZnPP-IX, significantly, but not completely, suppressed the anti-apoptotic effect of curcumin against H_2_O_2_. This data suggested that the anti-apoptotic effect of curcumin was probably attributed not only to the involvement of HO-1 but also to other elements. In addition, inhibition of PI3K/Akt pathway did not entirely reverse the curcumin-induced increase in HO-1 protein levels, suggesting that other PI3K/Akt-independent pathways are also involved in the effect of curcumin on HO-1. Moreover, a previous study showed that CO and bilirubin, products of heme metabolism by HO-1, exhibited a potent anti-apoptotic effect in doxorubicin-stimulated H9c2 cells [34], however, whether CO or bilirubin is involved in the cytoprotection afforded by curcumin is still unknown. Thus, experiments aimed at broadening our understanding of the more detailed mechanisms will be the subject of interest in future studies.

## Conclusions

Our results demonstrated that curcumin can protect H9c2 cells from H_2_O_2_-induced apoptosis and that such anti-apoptotic effect largely depends on the upregulation of HO-1 protein expression through the PI3K/Akt pathway. As a consequence, we speculate that curcumin, which exerts potential protection against oxidative stress-mediated apoptosis, might be used as a preventive and therapeutic agent for treatment of cardiovascular diseases associated with oxidative stress.

## Abbreviations

H_2_O_2_: hydrogen peroxide
HO-1: heme oxygenase-1
CC3: cleaved caspase-3
ROS: reactive oxygen species
CO: carbon monoxide
PKB: protein kinase B
PI3 K: phosphoinositide 3-kinase
ERKs: extracellular signal-regulated kinases
MTT: methyl thiazolyl tetrazolium
ZnPP-IX: Zine protoporphyrin-IX
DMSO: dimethyl sulfoxide
ATCC: American Type Culture Collection
